# Multivalent Carbohydrate-Lectin Interactions: How Synthetic Chemistry Enables Insights into Nanometric Recognition

**DOI:** 10.3390/molecules21050629

**Published:** 2016-05-13

**Authors:** René Roy, Paul V. Murphy, Hans-Joachim Gabius

**Affiliations:** 1 Pharmaqam and Nanoqam, Department of Chemistry, University du Québec à Montréal, P. O. Box 8888, Succ. Centre-Ville, Montréal, QC H3C 3P8, Canada; 2 School of Chemistry, National University of Ireland Galway, University Road, Galway, Ireland; paul.v.murphy@nuigalway.ie; 3 Institute of Physiological Chemistry, Faculty of Veterinary Medicine, Ludwig-Maximilians-University Munich, Veterinärstr. 13, München 80539, Germany; gabius@tiph.vetmed.uni-muenchen.de

**Keywords:** agglutinin, galectin, glycocluster, glycodendrimer, glycophane, glycoprotein, liposomes, oligosaccharides, sugar code

## Abstract

Glycan recognition by sugar receptors (lectins) is intimately involved in many aspects of cell physiology. However, the factors explaining the exquisite selectivity of their functional pairing are not yet fully understood. Studies toward this aim will also help appraise the potential for lectin-directed drug design. With the network of adhesion/growth-regulatory galectins as therapeutic targets, the strategy to recruit synthetic chemistry to systematically elucidate structure-activity relationships is outlined, from monovalent compounds to glyco-clusters and glycodendrimers to biomimetic surfaces. The versatility of the synthetic procedures enables to take examining structural and spatial parameters, alone and in combination, to its limits, for example with the aim to produce inhibitors for distinct galectin(s) that exhibit minimal reactivity to other members of this group. Shaping spatial architectures similar to glycoconjugate aggregates, microdomains or vesicles provides attractive tools to disclose the often still hidden significance of nanometric aspects of the different modes of lectin design (sequence divergence at the lectin site, differences of spatial type of lectin-site presentation). Of note, testing the effectors alone or in combination simulating (patho)physiological conditions, is sure to bring about new insights into the cooperation between lectins and the regulation of their activity.

## 1. The Third Alphabet of Life

**Figure 1:**
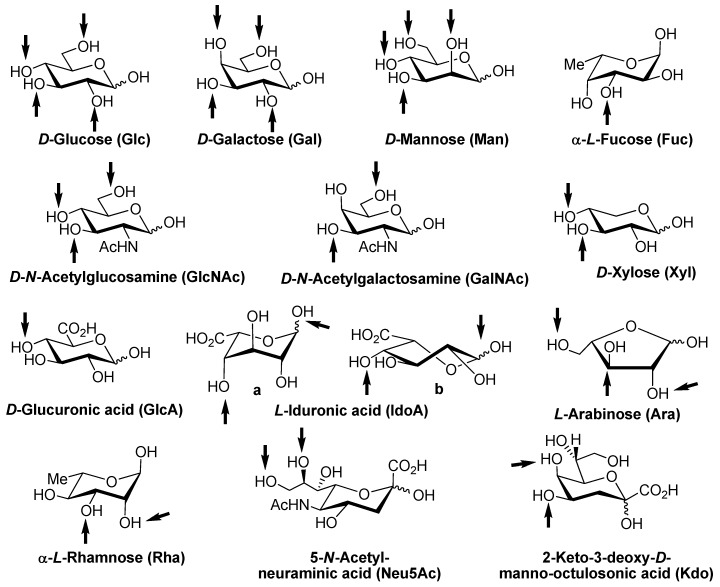
Illustration of the alphabet of the sugar language. Structural representation, name and symbol as well as the set of known acceptor positions (arrows) in glycoconjugates are given for each letter. Four sugars have l-configuration: fucose (6-deoxy-l-galactose), rhamnose (6-deoxy-l-mannose) and arabinose are introduced during chain elongation, whereas l-iduronic acid (IdoA) results from post-synthetic epimerization of glucuronic acid at C-5. The ^1^C_4_ conformation of IdoA (**a**) is in equilibrium with the ^2^S_0_ form (**b**) in glycosaminoglycan chains, where this uronic acid can be 2-sulfated. All other letters are d-sugars. Neu5Ac, one of the more than 50 sialic acids, often terminates sugar chains in animal glycoconjugates. Kdo is a constituent of lipopolysaccharides in the cell walls of Gram-negative bacteria and is also found in cell wall polysaccharides of green algae and higher plants. Foreign to mammalian glycobiochemistry, microbial polysaccharides contain the furanose ring form of d-galactose and also d/l-arabinose, then indicated by an italic “**f**” derived from the heterocyclus furan. The α-anomer is prevalent for the pentose arabinose, e.g., in mycobacterial cell wall arabinogalactan and lipoarabinomannan. β1-5/6-Linked galactofuranoside is present in the arabinogalactan and the α1-3/6 linkage in lipopolysaccharides (adapted from [15]).

Synergy, by teaming up chemistry with biosciences, can be expected to arise when the topic on which to apply complementarity of the disciplines is clearly defined. In principle, the chemical platform of life includes molecular alphabets, whose ‘letters’ are a toolbox to form ‘messages’ (or ‘words’) in the form of oligomers and polymers. “To an observer trying to obtain a bird’s eye view of the present state of biochemistry, or what is sometimes referred to as molecular biology, life may until very recently have seemed to depend on only two classes of compounds: nucleic acids and proteins” [1]. Their broad natural occurrence is shared by a third group, *i.e.*, glycans. Present in Nature as polysaccharides such as chitin, or as part of cellular glycoconjugates, *i.e.*, glycoproteins and glycolipids [2,3,4,5,6,7,8,9,10,11,12,13], their ubiquity and abundance have attracted interest to characterize rules of structural design, starting with the composition. This systematic work on material from pro- and eukaryotes step by step led to compiling the list of all individual building blocks, which form cellular glycocompounds. With the analogy to a molecular language in mind, this list constitutes the third alphabet of life, which complements the alphabets of nucleotides and amino acids (Figure 1). As is common for amino acids in proteins, substitutions such as phosphorylation or sulfation also broaden the range of letters for glycans. This use of the same principle of post-synthetic modifications underscores the equivalence of these alphabets with respect to their involvement in the flow of information from genes to cellular activities [14,15].

Drawing on textbook knowledge, the consideration of availability of several hydroxyl groups for building the glycosidic bond lets the special talent of carbohydrates to encode bioinformation become obvious. In contrast to the fixed connecting points of nucleotides and amino acids, two monosaccharides can be covalently linked to yield a groups of isomers: the anomeric position and linkage points are the sources of variability. What initially appeared as a deterrent to work with glycans, that is the enormous structural variety unsurpassed among biopolymers that so fittingly is expressed in the term ‘complex carbohydrates’, was then realized to be the basis for enabling high-density coding. Ironically, what at first impeded smooth progress turned out to be a means to put signals for different aspects of cell sociology into a minimum of space, an amazing functional dimension of glycans [14,15]. Due to the growing realization of this potential developing tools for chemical analysis of glycans on all structural levels [16] is crucial to delineate structure-activity relationships. That “in many cases the real role of biological glycoconjugate glycans is still unknown and, in many cases, we are unable to answer the question: why are proteins and lipids glycosylated?” [17] and that “on the whole, the role of many glycans remains obscure and the subject of speculation or controversy” [17] is no longer valid. In fact, the sequence of glycans, as in proteins or nucleic acids, gives shape to these biomolecules, seemingly minor alterations acting like switches between conformations [18,19,20,21,22]. Of note, the often encountered limitations to intramolecular flexibility of biorelevant oligosaccharides, combined with their enormous potential to engage in intermolecular recognition via hydrogen bonding, C-H/π-interaction and apolar complementarity, make them well-suited as binding partners. They are like molecular keys fitting into appropriate locks, and such receptors are present in Nature, as previously outlined in detail in this journal for lectins [23,24].

## 2. Lectins: Translators of Sugar-Encoded Information

Given the realized potential of carbohydrates to serve as a chemical platform for generating a large array of molecular signals, which defines the glycome, a functional implication could be expected if emergence of sugar receptor(s) is not a singular event in evolution. Strongly supporting the concept of significance of carbohydrate-protein binding, several families of proteins with this ability have developed, among them figuring prominently the lectins. They are separated from enzymes treating the bound glycan as substrate, immunoglobulins and transport proteins for free mono- or oligosaccharides [23,24,25]. In the case of animals and man, more than a dozen types of protein folds are capable to form sites to accommodate such ligands on the basis of mutual recognition (for a gallery of these lectins presenting folds and a snapshot of the molecular rendez-vous with a ligand, please see [26]), a sweet complementarity. Structurally, the architecture of the contact site spans the range from shallow groves to deep pockets, in certain cases recruiting the assistance of Ca^2+^ for structural organization or coordination bonds with hydroxyl groups of the sugar [26,27,28,29,30]. Physiologically, the architecture then determines the reactivity to glycans, generally with no or very limited promiscuity. From the perspective of the glycan, the same sugar epitope can become the target of several lectin types. As a means of implementing flexibility and responsiveness, these cellular signals can undergo a recoding without requiring neosynthesis. Enzymatic remodeling *in situ* can drastically alter a glycan’s reactivity its binding profile, opening the way to dynamic up- or down regulation of binding capacity. A classical example is the conversion of the hexasaccharide of the disialo-ganglioside GD1a to that of monosialo GM1 by a cell surface ganglioside sialidase (Neu3). Removal of one sialic acid moiety (for its structure, please see Figure 1) from ganglioside GD1a generates a functional marker. It signals differentiation of neuroblastoma cells *in vitro* and acquired responsiveness of activated effector T cells to a control by regulatory T cells, in both cases sensed exerted by an endogenous lectin [31,32]. The formation of a complex between the produced ligand, *i.e.*, the pentasaccharide of ganglioside GM1, and the lectin, *i.e.*, the homodimeric galectin-1, is the molecular trigger for a signaling cascade, which results in the final response (growth/activity inhibition, setting limits to tumor cell proliferation and T cell-mediated activity causing auto-immune diseases). This connection between the glycan and the cell sociology justifies to call the human lectin a translator of the sugar (ganglioside GM1)-encoded message.

**Figure 2:**
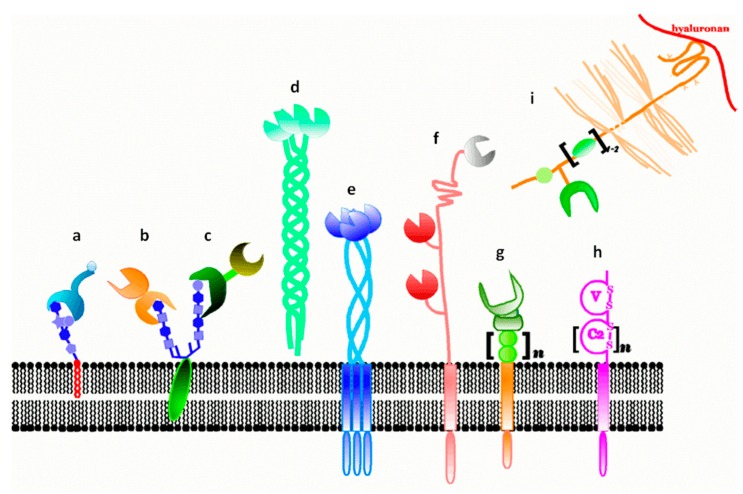
Illustration of the strategic ways that carbohydrate recognition domains (CRDs) in animal lectins are positioned to reach optimal ligand selection (for example, to separate self from non-self glycan profiles in innate immunity) and topological complementarity. From left to right, the CRD display in the three subtypes within the galectin family (chimera-type (**a**); proto-type (**b**) and tandem-repeat-type (**c**) arrangements binding to the saccharide portion of a ganglioside or a branched complex-type *N*-glycan without or with terminal α2,3-sialylation; please see also Figure 3), the presentation of CRDs (C-type or fibrinogen-like domain) in serum and surfactant collectins or ficolins (**d**) connected to their collagenous stalks and the non-covalent association of binding sites in transmembrane C-type lectins by α-helical coiled-coil stalks (**e**) (for example asialoglycoprotein and Kupffer cell receptors, the scavenger receptor C-type lectin, CD23, DC-SIGN or DC-SIGNR) are given. Similar to tandem-repeat-type galectins, the C-type family of lectins also has a branch of members with this design, *i.e.*, immulectins-1, -2 and -3. Next, the tandem-repeat display in the mannose-specific macrophage receptor (**f**) (also found on dendritic cells, hepatic endothelial cells, kidney mesangial cells, retinal pigment epithelial cells and tracheal smooth muscle cells) and the related C-type lectin Endo180 with eight domains as well as in the cation-independent P-type lectin with 15 domains is presented. Capacity for sugar binding is confined to only few domains as depicted. The occurrence of lectin activity for GalNAc-4-SO_4_-bearing pituitary glycoprotein hormones in the cysteine-rich domain, a member of the β-trefoil fold family with one (QxW)_3_ domain in the *N*-terminal section of the macrophage mannose receptor (amino acids 8–128), which is linked via a fibronectin-type-II-repeat-containing module to the tandem-repeat section, is also included into the schematic drawing for these lectins with more than one type of CRD per protein chain. Moving further to the right side; (**g**) the association of a distal CRD in selectins (attached to an epidermal-growth-factor (EGF)-like domain and two to nine complement-binding consensus repeats) or in the siglec subfamily of I-type lectins using 1–16 C2-set immunoglobulin-like units as spacer equivalents to let the CRD reach out to contact ligands and to modulate capacity to serve in *cis*- or *trans*-interactions on the cell surface is shown. The force-dependent alterations of the topological arrangement of the two distal domains in selectins accounts for catch bonds of selectins, a canonical immunoreceptor tyrosine-based inhibitory motif (ITIM) together with a putative tyrosine-based motif is frequently present in the intracellular portion of siglecs. C2-set domains linked to fibronectin-type-III repeats establish the extracellular section of the I-type lectins L1 and neural cell adhesion molecule (NCAM) (**h**). In the matrix, the modular proteoglycans (i) (hyalectans/lecticans: aggrecan, brevican, neurocan and versican) interact (i) with hyaluronan (and also link protein) via the link-protein-type modules of the *N*-terminal G1 domain (and an Ig-like module); (ii) with receptors binding to the glycosaminoglycan chains in the central region and (iii) with carbohydrates or proteins (fibulins-1 and -2 and tenascin-R) via the C-type lectin-like domain flanked by EGF-like and complement-binding consensus repeat modules (kindly provided by H. Kaltner) (from [38], with permission).

What is in general so intriguing about the recognition process with endogenous receptors is the exquisite selectivity of a tissue lectin (receptor) for distinct cellular glycoconjugates (in terms of the scaffold and its glycosylation). As it turned out, often only few glycoproteins or glycolipids from the complexity on cells are binders, and lectin reactivity is temporally and spatially tightly regulated. Modulations of glycoconjugate synthesis, routing and/or degradation and of the structure of the presented glycan(s) open ways to make cells specifically prepared for carbohydrate-protein recognition. To give a further biomedically relevant example, the tumor suppressor p16^INK4a^ is the master regulator for a glycan-based effector route to drive pancreatic carcinoma cells into programmed cell death: the underlying caspase-9 activation is achieved by clustering of α_5_β_1_-integrin and galectin-1 on the cell surface, a process favored by their concerted upregulation and the increase of galectin-1-suited integrin glycosylation through reducing α2,6-sialylation of its *N*-glycans [33,34]. That availability of an antagonist of galectin-1 in this context, *i.e.*, galectin-3, is downregulated [35] lays open intricacies of fine-tuning this effector pathway. In addition to the direct contact possible and the complementarity between the cognate glycan determinant and the lectin, factors of spatial presentation, too, will most likely matter. Multivalency of glycans, achieved by branching or tight arrangement in a microdomain, also artificially established by a lectin and synthetic ligands [36], facilitates fractional high-affinity binding in the nM (and even below) range for galectins [37]. To give research to unravel the molecular origin of the selectivity of matchmaking direction, affinity regulation has been formally assigned to work on six levels [30,38]. After the target identification, the endogenous lectin in this case acts as a cross-linker of a glycoprotein, thus turning attention to the modes of structural design of tissue lectins are relevant for the outcome. As glycan structures vary in terms of branching and repeats, per glycan and per glycoconjugate, various modes are realized to present a carbohydrate recognition domain (CRD) (Figure 2). Looking at the mentioned galectin-1 (bottom, left), it is a homodimer of two identical CRDs, ideal for cross-linking its counter receptor(s) to elicit the intracellular signaling cascade.

**Figure 3:**
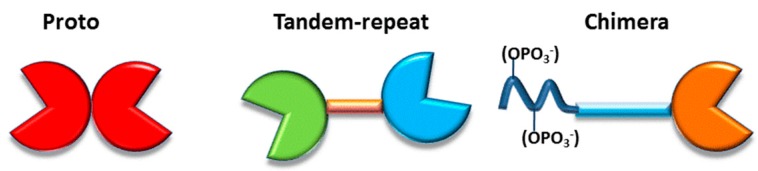
The three basic types of modular design of galectins, as given in the context of other lectins in Figure 2. Proto-type proteins (e.g., galectins-1, -2 and -7) are non-covalently associated homodimers, the vertebrate tandem-repeat-type galectins are formed by two different CRDs connected by a linker (length can vary by alternative splicing and between species) and the chimera-type galectin-3 has an *N*-terminal tail composed of a peptide with site(s) for Ser phosphorylation and Gly/Pro-rich sequence repeats enabling self-association in the presence of multivalent ligands.

Looking through the different sections of Figure 2, and through its legend presenting further details on the respective lectins, it becomes clear that the topology of CRD presentation has gained a large degree of diversity, and there is more. Detailed analyses within lectin families based on database mining have underscored that the course of evolution has led to complexity on several levels: gene diversification in terms of variations of the number of genes in each family by duplications/losses and of the individual sequences, even among related species [39,40,41,42,43,44]. In overview, covalent and non-covalent modes of linking CRDs as well as the connection of CRDs with other types of modules are frequent among lectins, signaling a fundamental functionality to be disclosed by thorough structure-activity investigations. Here, using bioinspired synthetic ligands as sensors or glycoclusters as molecular rulers offers a promising perspective. To depict principles of this line of research we here describe instructive examples selected from work on a class of adhesion/growth-regulatory lectins. How carbohydrate chemistry can advance our level of understanding of aspects of how selectivity is achieved is thus examined for biomedically relevant effectors, two already mentioned above. These proteins are referred to as galectins (please see Figure 2, left part and Figure 3 for their modes of structural design in vertebrates).

## 3. Galectins: Definition and Structures

To qualify to become a canonical member of this family a protein must satisfy the following criteria:

(i) to have a CRD with a β-sandwich fold, first described in this field for the leguminous lectin concanavalin A and illustrated in the Gallery of Lectins [27], is a compact structural platform consisting—in the case of human galectin-1—of two antiparallel β-sheets of five to six strands, respectively,
(ii) to bind compounds from the group of β-galactosides and
(iii) to share a sequence signature (mostly the amino acids in contact with the ligand, especially a central Trp for C-H/π-interaction with the B-face of galactose) [26,43,45,46,47].

**Figure 4:**
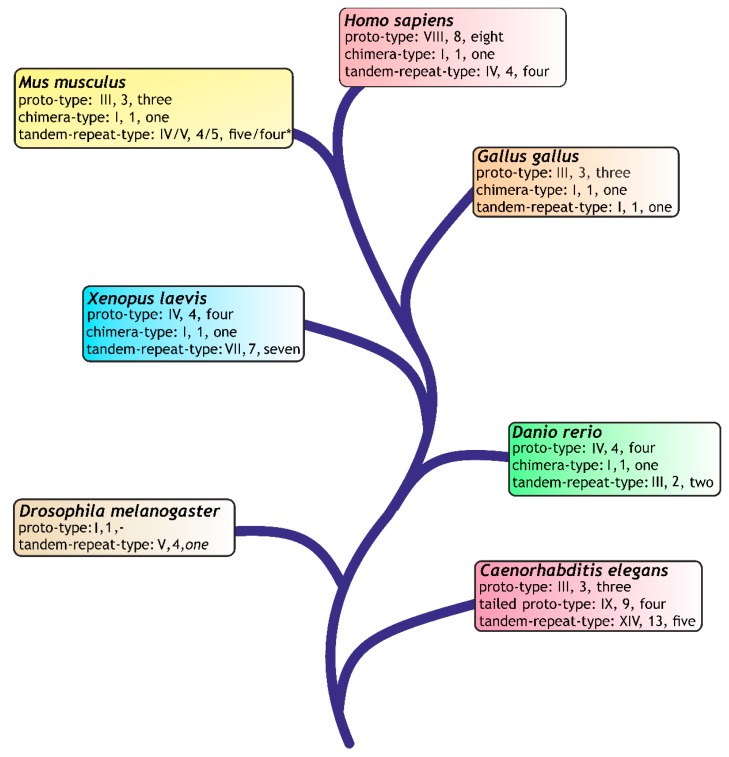
Canonical galectins (according to the classification given in Figure 2) in model organisms on the level of gene (Roman number), transcript (Arabic number) and protein (numerical information). For details, please see [42,43].

In vertebrates the CRD is commonly presented in three topological modes given in Figure 3, associated with a tail consisting mostly of non-triple helical collagen-like repeats suited for aggregation (chimera type), as a non-covalently associated homodimer (proto type) and as a covalently linked heterodimer (tandem-repeat type). Before synthetic work to prepare binders for galectins is outlined, an overview of diversity of galectin representation among species is given. Computational mining of databases, together with experimental validation of active genes, accounts for the phylogenetic tree that summarizes the number of galectins in selected species (Figure 4). Reflecting the dynamics of the evolutionary process, the galectin display is not constant between species, an important fact precluding simple extrapolations and calling for thorough analysis of each individual protein. Equally important, galectins likely form a network of members of each group (to give a clinically relevant example, colon cancer cells express most tested galectins, responsive to differentiation and providing prognostic information [48,49], posing the challenge to define properties of the individual proteins and cooperation/antagonism *in situ*.

**Figure 5:**
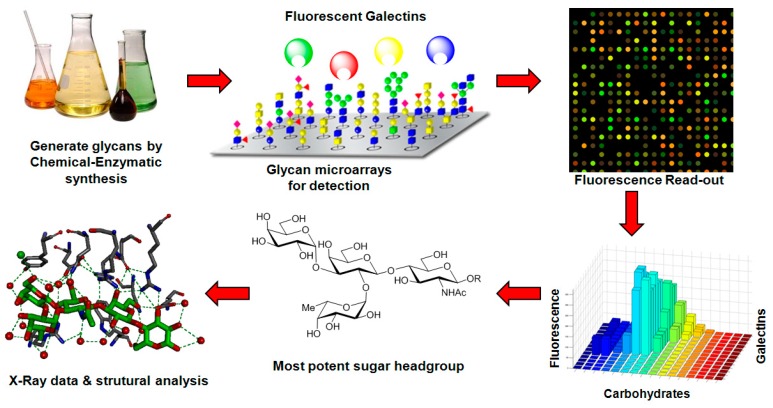
Sequence of steps to identify high-affinity carbohydrate ligands for galectin binding. Galectin interaction with the most potent binders based on this screening can then be studied further on the structural level.

**Figure 6:**
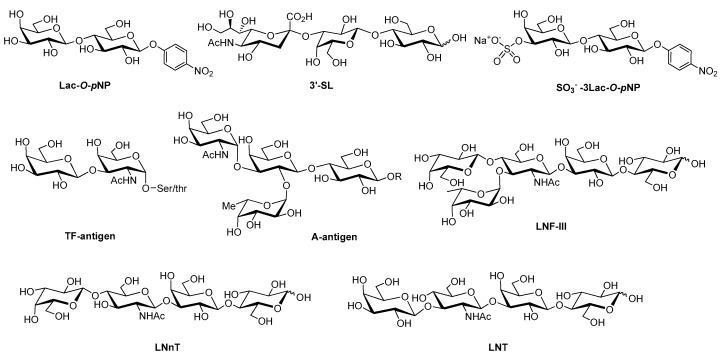
Representative structures of galectin ligands.

To start characterizing the ligand specificity, synthetic chemistry is invaluable due to its ability to make lead compounds available for affinity testing (for a compilation of experimental approaches to characterize lectin activity, please see Table 1 in [26]). Due to the advantage of requiring only small amounts of lectin and ligand on a multi-assay scale, glycan microarrays have become instrumental for comparative analysis, setting the stage for the structural follow-up studies (for a flow scheme, please see Figure 5) [50,51,52,53,54]. This and other glycan-based techniques delineated the grading of bioactivity of diverse glycans, teaching the lesson that various structural changes, e.g., introduction of sialylation or sulfation (as mentioned above) and extension to oligosaccharides with branching and sequence repeats, can matter markedly for distinct members of the family (for examples, please see [55,56,57,58,59,60,61,62,63,64,65]). With lactose as reference, significant affinity enhancements were found for the saccharides given in Figure 5, for example with the synthetic *p*-nitrophenyl lactoside, a classical test case studied up to now, recently by NMR spectroscopy using ^15^N-labeled galectin-1 [66]. These structures, at the same time, establish a platform to proceed to address the issue of selection. One preferred galectin ligand, as depicted in Figure 6 (bottom), is a repeat of the disaccharide [67]. Questions to be answered are whether and to what extent homologous proteins have different binding properties and whether a target-specific blocking might be accomplished.

**Figure 7:**
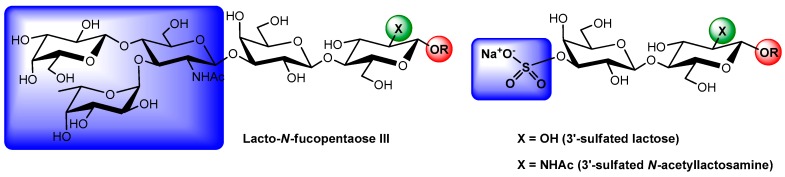
Comparison of structures of LNF-III (**left**) and 3′-sulfated lactose derivatives (**right**) that bind almost equally well to human galectin-8N. Suggested additional modifications of the sugar head groups together with their substituents, which might increase the affinity of glycans toward galectins, while limiting synthetic efforts towards this achievement, are highlighted.

**Figure 8:**
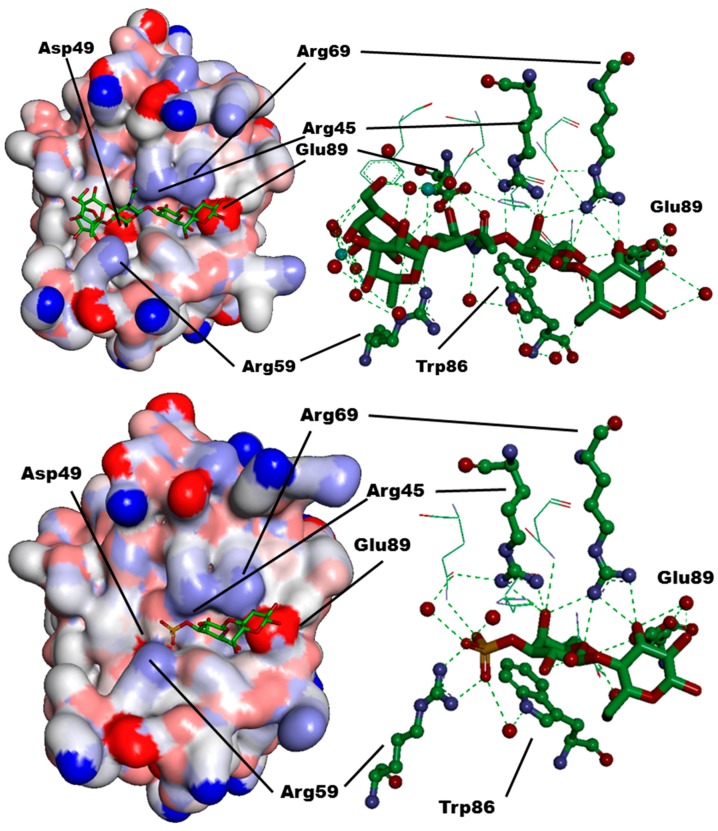
Human galectin-8N (Connolly surfaces and binding sites) loaded with lacto-*N*-fucopentaose-III (**top**) and 3′-sulfated lactose (**bottom**) (PDB 3AP9).

In that case, a high-affinity and specific carbohydrate derivative would need to be identified for each galectin. Because the CRDs of the galectins are rather conserved, this represents a formidable synthetic challenge. However, if one takes the *N*-terminal CRD of galectin-8 as an example, distinct preferences have indeed developed [68,69,70,71,72,73,74]. Loaded with either LNF-III (Figure 7 left) or 3′-sulfated lactose (Figure 7 right), the structures of the complexes and the intimate interactions (shown in Figure 8) reveal that it becomes conceivable that achieving selectivity gains is not an insurmountable task. This example clearly demonstrates that suitably functionalized carbohydrate derivatives can even take the place of rather complex glycans. Indeed, already a rationally derivatized disaccharide can offer a substantial selectivity gain, despite the overall rather conserved contact pattern of galectins for lactose (Table 1).

**Figure 9:**
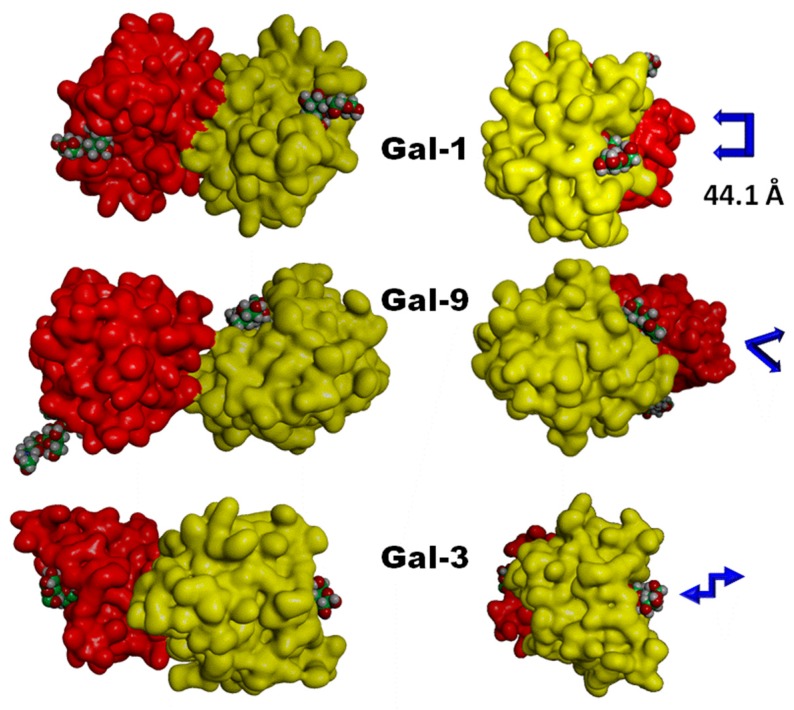
Relative spatial orientation of CRDs of galectins, which can be differently responsible for intra/inter-cellular cross-linking (**left**: face view; **right**: lateral view). Blue arrows point the positioning of two CRDs.

In view of Figure 3, the structural design of the galectins will then also be an incentive for tailoring spatial aspects of binding partners, to optimize selectivity and to unravel structure-activity relationships, as the protein, too, can be tailored, for example engineering new homodimers (Figure 9). Strategically, the ligand structure can be modified for optimal fit at different sites, with the aglycone, as the case of *p*-nitrophenyl, as starting point, a strategy defined as subsite-assisted binding [75].

## 4. Tailoring Monovalent Ligands for Selectivity

In chemical terms, each structural part of the ligand can be examined for its contribution to selectivity. Thus, the aglycone (in glycoclusters the linker to the scaffold), the atom at the glycosidic linkage and the sugar (sequence and substitutions) are subject to changes on the drawing board, providing interesting compounds to be synthesized and tested.

### 4.1. Modifications of the Aglycone

**Scheme 1:**
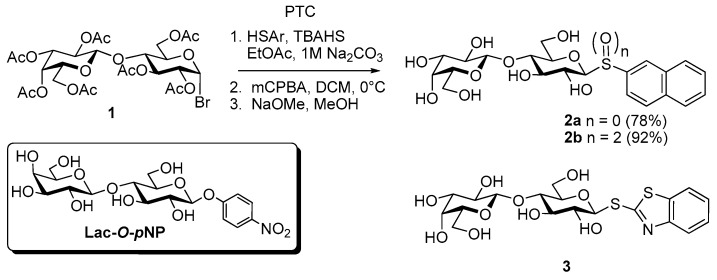
Synthetic strategy leading to *O*- or *S*-lactosides under phase transfer-catalyzed (PTC) conditions.

The classical example is the aromatic extension by a *p*-nitrophenyl group, reported for galectin-1 (formerly called galaptin) [57,58]. In detail, a 2.5-fold enhancement was seen by adding this group to lactose [57], and even a 19-fold enhancement was observed when it was used as an extension to galactose [58]. That alterations in the aglycone part have the capacity to generate contributions to selectivity has been delineated by testing a series of β-lactosides with aromatic substituents [76]. β-Naphthyl and 2-benzothiazolyl proved especially active, in terms of affinity to galectin-3 relative to galectin-1 in isothermal titration calorimetry and in comparative inhibition assays [76,77,78]. As illustrated in Scheme 1, the synthesis of the lactosides started from readily available peracetylated lactosyl bromide **1**, which was obtained in quantitative yield by treatment of peracetylated lactose with HBr in AcOH. As expected, the phase transfer-catalyzed (PTC) nucleophilic displacements of lactosyl bromides by the aryl alcohols or aryl thiols occurred with complete anomeric inversion to afford exclusively the corresponding β-glycosides in good to excellent yields (78% for **2a** and 75% for **3**) [76]. They were ready to be evaluated for ligand activity with galectins after Zemplén de-*O*-acetylation (NaOMe, MeOH). With β-napthyl thioglycoside such as the peracetylated derivative **2a** in hand, synthesis of anomeric sulfones by oxidation of the sulfide groups using *m*CPBA (2.1 equiv, CH_2_Cl_2_) generated sulfones **2b** (92%) after de-*O*-acetylation. Most *O*-aryl lactosides were estimated as being 3-fold better than lactose in activity assays, thus underscoring a preference for aromatic aglycons. What’s more, the data reveal this region to be of conspicuous relevance for selectivity [76,77,78].

On the structural level, the *O*-3 hydroxyl group of the glucose moiety of lactose is the main contact of this part of lactose with galectins, as first detected by chemical mapping β-methyl lactoside derivatives (deoxy and fluoro) [79]. Semi-empirical calculations correlated well with the chemical modifications done at the anomeric position with the effect on charge density at *O*-3 of the glucoside residue of lactose. The *O*-3 electron density calculations were made on the lactoside derivatives evaluated on galectin-1 in the study [80,81,82]. Electron-withdrawing aglycones, for example the sulfone moiety in **2b**, decreased the *O*-3 electronic densities and favored interactions with Glu71, which was accounted for substantially by hydrogen bonding with the human lectin [83]. Hence, a direct correlation exists between the inhibitory potencies and the charge density at *O*-3 calculated on electrostatic potential. Lac-*O*-*p*NP has a theoretical electron density of −0.346 compared to that of its o-nitrophenyl analog (−0.322). This difference of electron density could explain the relative inhibitory potency variation on galectin-1. Compound **2b**, having the lowest *O*-3 electron density, also has the highest inhibitory potency [76]. Among the compounds tested by isothermal calorimetry (ITC), benzothiazole **3** showed the best thermodynamic performance (ΔG of −6.7 kcal/mol; Ka 65 × 10^−3^ M^−1^) for galectin-3 [76].

**Scheme 2:**
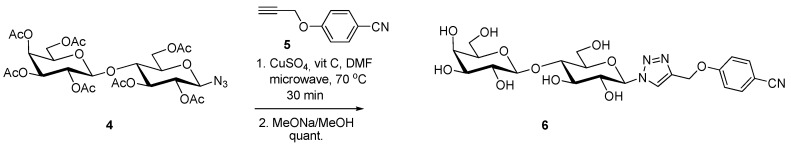
Representative synthesis of regioselectively 4-substituted 1,2,3-triazole lactoside **6** using copper-catalyzed azide–alkyne cycloaddition (CuAAC, click chemistry).

When assaying derivatives harboring a 4-substituted 1,2,3-triazole unit, derived from lactosyl azide **4** using copper-catalyzed azide–alkyne cycloaddition (Scheme 2) to afford heteroaryl lactoside **6** in essentially quantitative yield after Zemplén deprotection, the potential of substitution at this site was reinforced [66].

### 4.2. Modifications at the Glycosidic Linkage

**Scheme 3:**
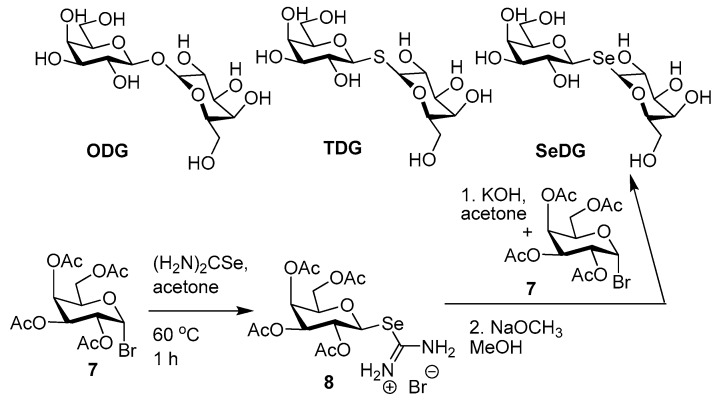
Structures of *O*-digalactoside (ODG) and its *S*- and *Se*- mimics (TDG and SeDG), with synthesis of SeDG.

The introduction of S, Se or C atoms into the glycosidic linkage makes the glycoside resistant to glycosidases. Also, stereoelectronic aspects, bond angles and flexibility are altered [84,85,86]. Testing the S/Se-bridged digalactosides (TDG, SeDG), rather small discriminatory effects were noted whereas dithio/seleno bridging was unfavorable for galectins [87]. However, S/Se-digalactoside activity to a plant toxin opens the possibility for avoiding cross-reactivity, if desiring to block the toxin [87]. The synthesis of the SeDG is summarised in Scheme 3. The peracetylated galactosyl bromide is treated with selenourea in acetone to generate a salt. Production of the selenolate anion from this salt followed by reaction with **7** gave SeDG. A related approach, using thiourea, gives TDG via glycosyl thiol intermediates, which are generally useful in *S*-glycoside synthesis.

Proceeding from the reducing end, the next part that can be altered is the sugar headgroup. Here, a brief look at Figure 6 indicates that certain positions are preferred sites for increasing reactivity to galectins, inspiring exploitation.

### 4.3. Modification at Bioinspired Positions

The synthetic extensions of disaccharides to natural oligosaccharides has enabled to map contact sites in the CRDs beyond lactose. An example for the synthesis of relevant structures for galectins is the addition of a fucose moiety in an α1,2-linkage to lactose, constituting the histo-blood group H-trisaccharide, with general principles of oligosaccharide synthesis outlined in [88]. The synthesis of oligosaccharides enables generation of stereochemically pure and homogeneous substances for evaluation of their binding to lectins. It also enables the incorporation of these substances onto microarrays for identification of ligands for lectins (Figure 5). Chemical synthesis of glycoside bonds relies on having both suitably protected donors and acceptors available. The synthesis of 2′-fucosyl lactose exemplifies an approach to generating a lactos derivative [89]. Matta and coworkers have shown that lactose can be converted to **9** via acetonide formation and benzoylation [90] and used this in a synthesis of 2′-fucosyl lactose. This leaves the 2′-OH free to be a nucleophile in a glycosidation reaction. In a variation of Matta’s synthesis, a benzylated thiofucopyranoside **10** was shown to act as a donor using Crich and Smith’s activation protocol [91]. The use of benzyl protecting groups, which do not participate in the glycosidation reaction, ensure that the required α-stereoisomer **11** is formed after activation of the thioglycoside. Most likely, the undesired β-anomer would have resulted if acylated protecting groups had been used, unless anomerisation could occur or be induced *in situ* [92]. Subsequent removal of the benzoyl and acetonide protecting groups generates **13** and, finally, 2′-fucosyl lactose is obtained by hydrogenolytic removal of the benzyl groups.

**Scheme 4:**
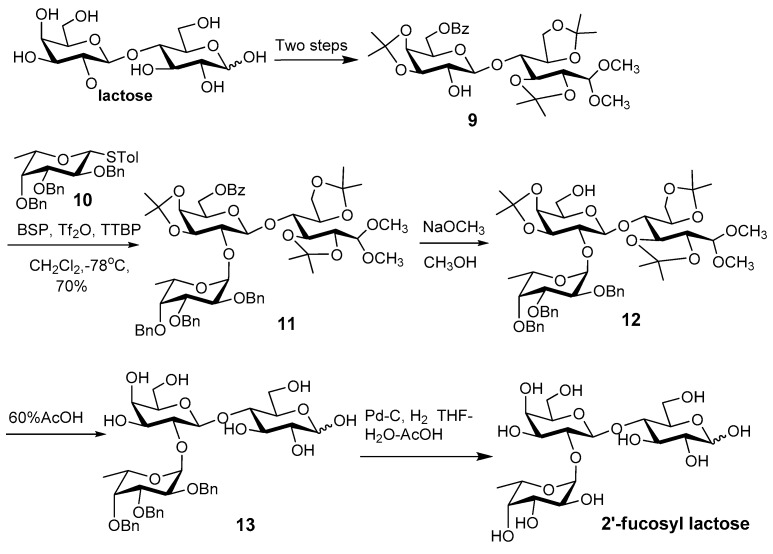
Example of oligosaccharide synthesis to produce naturally occurring galectin ligands.

**Scheme 5:**
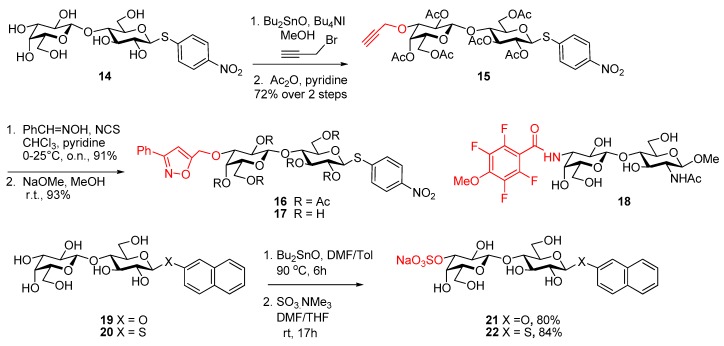
Chemical modifications of lactosides at both the aglycones and at the 3′-position.

As noted above with respect to Scheme 4, potent binding partners for galectins present distinct substitutions at the *N*-acetyllactosamine core, for example the α1,3-linked *N*-acetylgalactosamine residue, which turns the histo-blood group H-type trisaccharide to the A-type tetrasaccharide. Such a site for a substitution is referred to as a rational design based on structural information (bioinspiration) Scheme 5 documenting typical reaction sequences toward the production of compounds following this bioinspiration.

In general, irrespective of the aglycone residues on unprotected β-d-lactopyranosides, it is possible to use the well-known behavior of tin-ether or tin-acetal activation that greatly and regioselectively potentiate the nucleophilicity of the *O*-3′ oxygen to modify this particular position. For example, lactoside **14** can be selectively propargylated to give compound **15** in very good yield after acetylation [76]. The propargyl ether of **8** was then treated by a [1,3]-dipolar cycloaddition [93] between the alkyne and nitrile oxide, generated *in situ* from benzaldehyde oxime and *N*-chlorosuccinimide (through intermediate benzhydroximoyl chloride) to afford **16** in 91% yield, which after Zemplén deprotection provided the desired ligand **17** almost quantitatively. An analogous strategy was used to give the corresponding 3′-sulfated *O*- and *S*-naphthyl lactosides **21** and **22** from **19** and **20**, respectively. It is also worth noting that the above chemical sequences provided compounds reactive with galectins-1 and -3 in a much faster route than the one previously described for the synthesis of **18** showing a K_D_ of 320 nM against galectin-3 [94].

**Figure 10:**
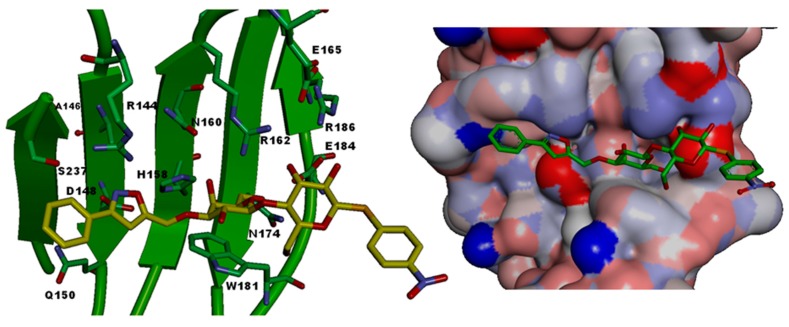
Schematic representation of the CRD site of human galectin-3 (PDB 3AYE) showing all amino acids involved as per Table 1 and interacting with compound **17** (docked in by superposition with natural lactose ligand); left Connolly surface representation with red surface (anionic) and blue surface (cationic amino acids).

To initiate to explain its marked reactivity to galectins-3, -4 and -8, the isoxazole lactoside **17** was docked into the active site of human galectin-3. Possible interactions with all 13 amino acid residues listed in Table 1 can thus be monitored. It can be clearly seen that **17** may form strong hydrogen bonding with Asp148, H158, and possibly the key remote Arg144 with the isoxazole moiety (Figure 10). Evidently, thorough study of structure-activity relationships (SARs) offers remarkable potential to understand selectivity and push an enhancement to its inherent limits.

In summary, a binding partner of a (ga)lectin has been formally separated into distinct sections. The advantage of this approach is that any structural change introduced by synthesis can be evaluated and optimized. Combinatorial integration can then fully realize the potential of tailoring the monovalent structure. Of note, activity testing should include the natural panel of lectins (of the same family and of other families sharing the same nominal glycan specificity) to preclude premature conclusions on selectivity. Hence, keeping an eye on the size of the lectin network, as illustrated in Figure 4, and on cross-reactive activities is essential. With this part done, the next source of inspirations for tailoring synthetic binders of lectins comes from the natural presentation of glycans as multivalent clusters up to microdomains. Affinity and selectivity are increased when properly taking spatial factors of the presentation into account, the fine work on the glycoside cluster effect with pioneering experiments on the hepatic asialoglycoprotein receptor, a C-type lectin shown schematically in Figure 2, and synthetic glycoclusters serving as excellent precedent [95,96].

## 5. Tailoring Spatial Aspects for Selectivity

A wide variety of scaffolds can be used to present glycans as bioactive ligands, generating glycoclusters or glycodendrimers [97,98,99,100,101]. Of course, any favorable structural feature found on the level of testing monovalent compounds can now be implemented. The following schemes can thus be considered as examples tested with galectins, starting with a selection of bivalent compounds.

**Scheme 6:**
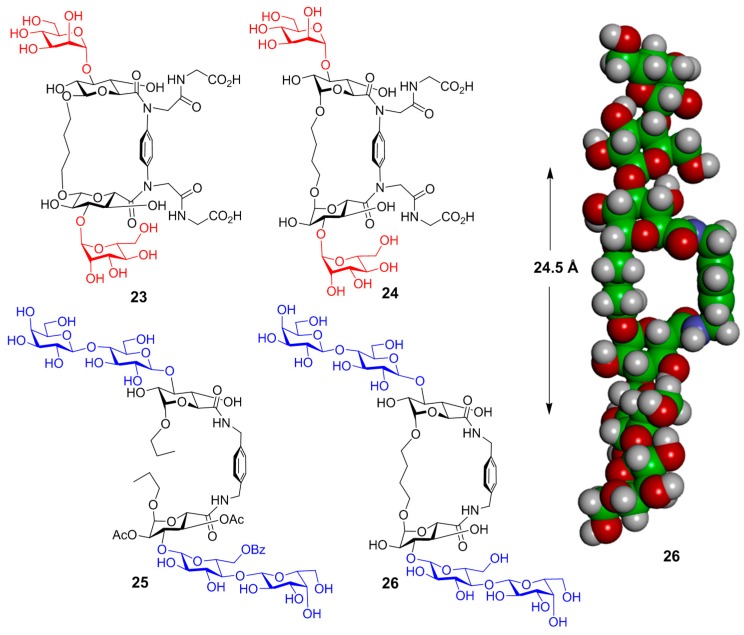
Structures of bivalent glycoclusters based on glycophane and an acyclic variant. Space-filling model of **26** is shown on right with distance between galactose anomeric carbon atoms indicated.

Macrocyclic scaffolds derived from glucuronic acid and phenylene-1,4-diamine (glycophanes; [102,103]) had been used as fragments in building bivalent mannosides **23** and **24** (Scheme 6) [104]. This inspired the use of such chiral scaffolds based on *p*-xylylenediamine to prepare structurally defined bivalent lactoside **26** [105]. Compound **26** is considered rigid when compared with **25**. Molecular modeling can be used to investigate distance profiles between galactose headgroups in such bivalent compounds. The use of macromodel for example, indicated that the intergalactoside distance in **26** was 24.5 Å. While both **25** and **26** showed similar affinity for galectin 3 in a solid phase binding assay, the glycophane **26** was >10-fold more potent than **25** towards the truncated galectin 3.

**Scheme 7:**
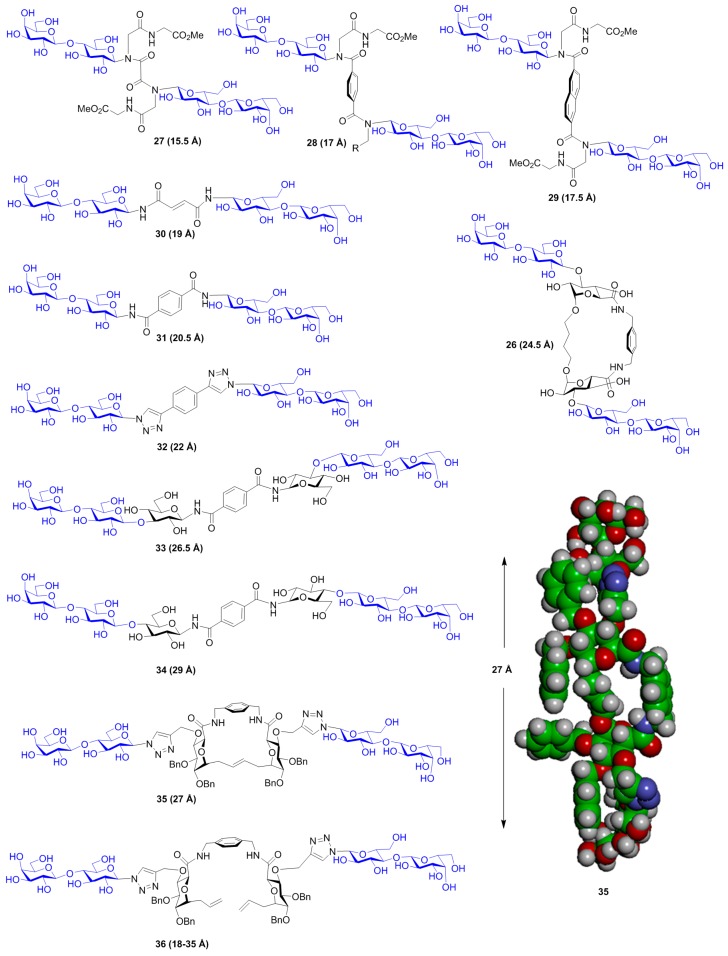
A panel of synthetic lactose-presenting bivalent compounds with inter-galactoside distances are shown. Various types of scaffolds have been used including secondary and tertiary diamides, diglycosyldiamides, ditriazoles and glycophanes.

Various different frameworks (scaffolds) can be incorporated in bivalent glycocluster design and synthesis and a selection are shown in Scheme 7 [105,106,107]. While topology within this series can be considered the same (*i.e.*, they are all bivalent), the spatial relationships are different, allowing this property to be investigated through bioassays. Interactions of the scaffold with the lectin that may contribute to, or hinder, binding cannot be discounted given, for example, activity observed for compounds such as **6** (Scheme 2). In the series shown the distances between the galactose residues can vary from 15.5 Å to 35 Å, as estimated by molecular modeling. A variety of scaffold types have been investigated including both tertiary (**27**–**29)** and secondary (**30**, **31**) diamides, diglycosyldiamides (**33**, **34**), ditriazoles (**32**) and the previously mentioned glycophane **26** together with a *C*-glycosyl version **35** and a more flexible variant of the latter **36**. Highlights from biological study of such a series include observations that the tertiary diamide **27** was 5-fold more selective for proteolytically truncated galectin-3 (complete removal of the non-CRD sections, shown in Figure 3) than **26** [104] and that the more flexible glycocluster **36** showed 100-fold selectivity for the chicken galectin-3 compared with chicken proto-type galectin-1A [106].

**Scheme 8:**
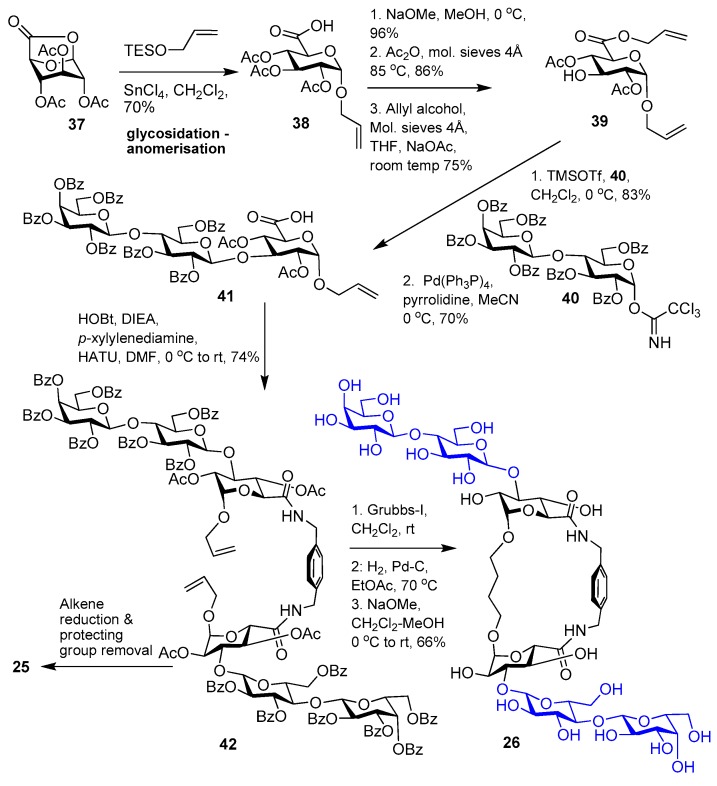
Synthesis of glycophane **26** and the acyclic analogue **25**.

The synthesis of **25** and **26** is summarised in Scheme 8 [104]. The glycosidation reaction of 1,6-anhydro donor **37** with ^1^C_4_ conformation, which is more reactive than a related donor with ^4^C_1_ conformation, occurs in the presence of triethylsilyloxyprop-1-ene to initially give a β-glycoside but this species undergoes rapid *in situ* anomerisation in the presence of SnCl_4_ to give the α-glucuronide **38** [108,109]. This was converted to **39** by removal of the acetate protecting groups followed by an acetylation reaction that gave a 6,3-lactone intermediate and subsequent reaction of this with allyl alcohol gave **39**. The glycosidation reaction between benzoylated trichloroacetimidate **40** and **39** and subsequent removal of the allyl ester using Pd(0) gave **41**. Next the coupling of acid **41** with *p*-xylylenediamine gave **42**. Ring closure metathesis using the Grubbs-I catalyst followed by alkene reduction and acyl group removal gave glycophane **26**, whereas alkene reduction and deacylation under similar conditions gave **25**. The use of the benzoyl protecting groups in **40** was important as the analogous per-*O*-acetylated donor gave none of the desired glycoside but only an orthoester, which can be observed in the course of glycosidation.

**Scheme 9:**
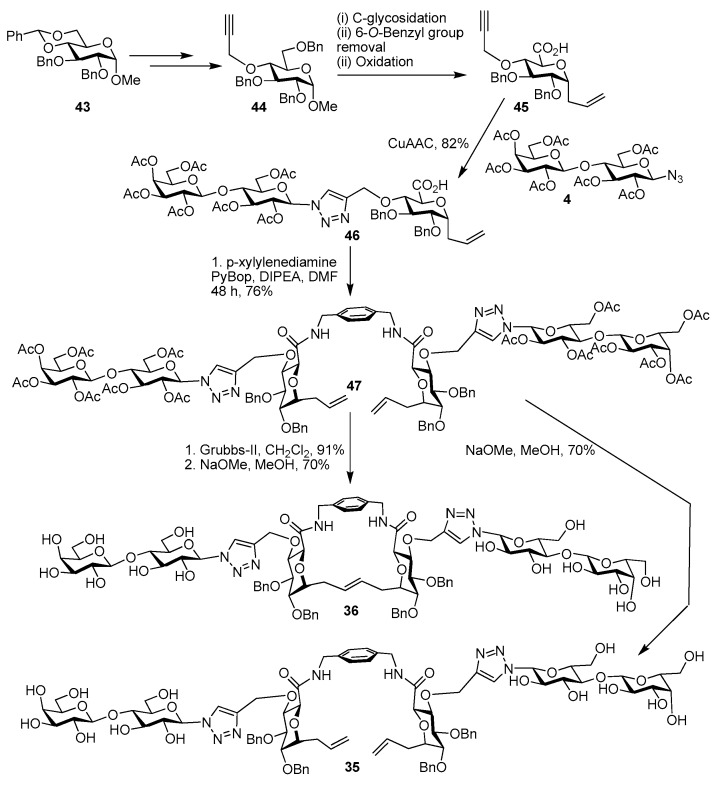
Synthesis of glycophane **36** and the acyclic analogue **35**.

Synthesis of **35** with the *p*-xylylenediamine based scaffold is shown in Scheme 9 [106]. The propargyl derivative **44** was first prepared from benzylidene **43** via a partial reductive cleavage of the benzylidene followed by 4-*O*-propargylation. Next formation of the *C*-glycosyl derivative **45** was carried by anomeric allylation [110], followed by selective removal of the 6-*O*-benzyl group and subsequent oxidation. The copper (I)-promoted triazole formation with azide **4** gave **46**. Subsequent coupling to *p*-xyxylenediamine followed by deacetylation gave **36**, whereas ring closure metathesis using Grubbs-II catalyst followed by de-*O*-acetylation gave **35**.

**Scheme 10:**
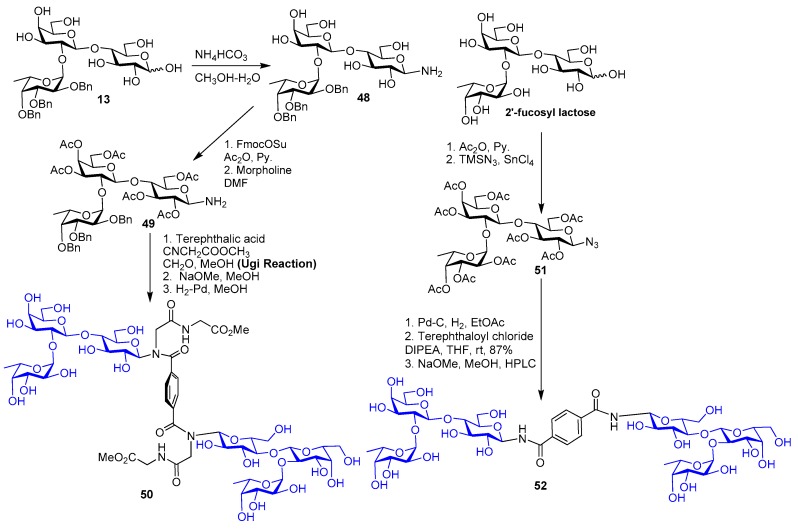
Synthesis of diamides with the histo-blood H trisaccharide headgroup.

**Scheme 11:**
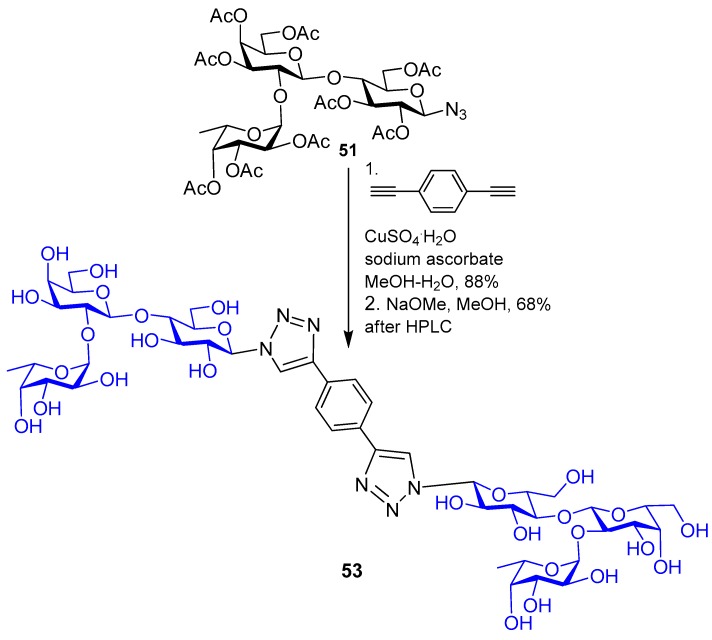
Synthesis of the bivalent compound **53**.

The chemistry shown in Scheme 10 exemplifies an approach to the synthesis of diamides where 2′-fucosyl lactose headgroups are incorporated [107]. Diamides can be prepared from corresponding glycosylamines such as **49**. The Ugi reaction is a four component reaction involving amine, acid, aldehyde and isocyanide that is suitable for tertiary amide generation. When a diacid such as terephthalic acid is used with **49**, it efficiently gives the divalent glycocluster **50** after protecting group removal. Alternatively, reduction of the azide **51** in the presence of Pd-C and hydrogen gave a glycosyl amine and subsequent coupling of this with terephthaloyl chloride and then deacetylation gave diamide **52**. The secondary amides in **52** prefer a *trans*-arrangement whereas those in **51** prefer *cis* and this difference gives rise to different spatial arrangements between the carbohydrate headgroups and can account for differences in preferences for lectins. The direct copper-promoted triazole formation from **51**, prepared from 2′-fucosyl azide (Scheme 11), and subsequent protecting group removal gave ditriazole **53**. This latter compound showed high affinity (IC_50_ = 8 μM) for galectin-4 and was ~6-fold more selective to this lectin than **52** [107].

**Scheme 12:**
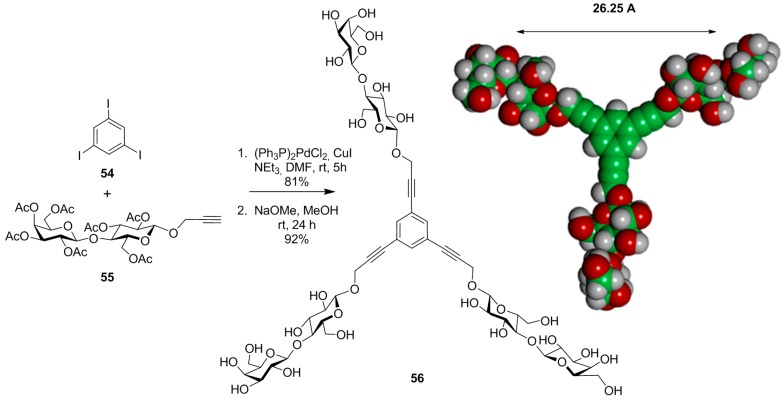
Trivalent lactose-presenting cluster **56** with equivalent inter-galactoside distances was obtained using the core **54** and 2-propynyl lactoside **55**. It showed remarkable reactivity with galectin-3 [111].

**Scheme 13:**
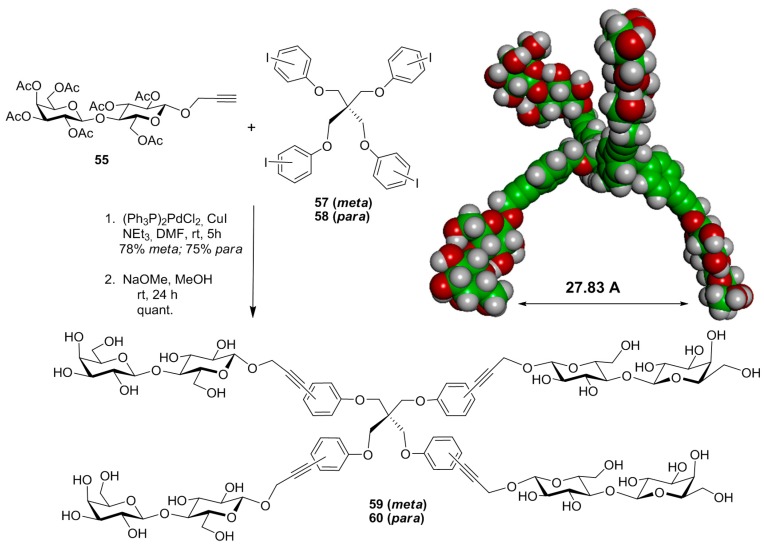
Tetrameric lactoside clusters **59** and **60** with different inter-galactoside distances prepared using palladium-catalyzed cross coupling Sonogashira reactions.

Valency increases give rise to new glycocluster topologies and can be taken to the level of tri- and tetravalent glycoclusters, as shown next with notable enhancements for the chimera-type galectin-3 [111]. Synthetically, 2-propynyl lactosides were conjugated under Sonogashira palladium-catalyzed cross-coupling conditions with triiodobenzene and suitably derivatized pentaerythritol cores (Scheme 12 and Scheme 13). Starting with 2-propynyl lactoside **55**, a series of tri- and tetra-valent derivatives **56**, **59**, and **60** were thus prepared using a common general protocol ((Ph**_3_**P)**_2_**PdCl**_2_**, DMF:Et**_3_**N (1:1, *v*/*v*), CuI, rt, 3–5 h). The simultaneous Sonogashira cross-coupling of **55** to 1,3,5-triiodobenzene **54** (Scheme 12) and to pentaerythritol tetrakis(*m*- and *p*-iodobenzyl)ether **57** or **58** (Scheme 13) afforded an efficacious entry into this family of carbohydrate clusters **56** (trimer 80%), and tetramers **59** (*meta*) and **60** (*para*) in 78 and 75% yields, respectively. Complete de-*O*-acetylation of these clusters was uneventful, resulting in freely water-soluble clusters **56**, **59**, and **60** in more than 90% yields.

**Scheme 14:**
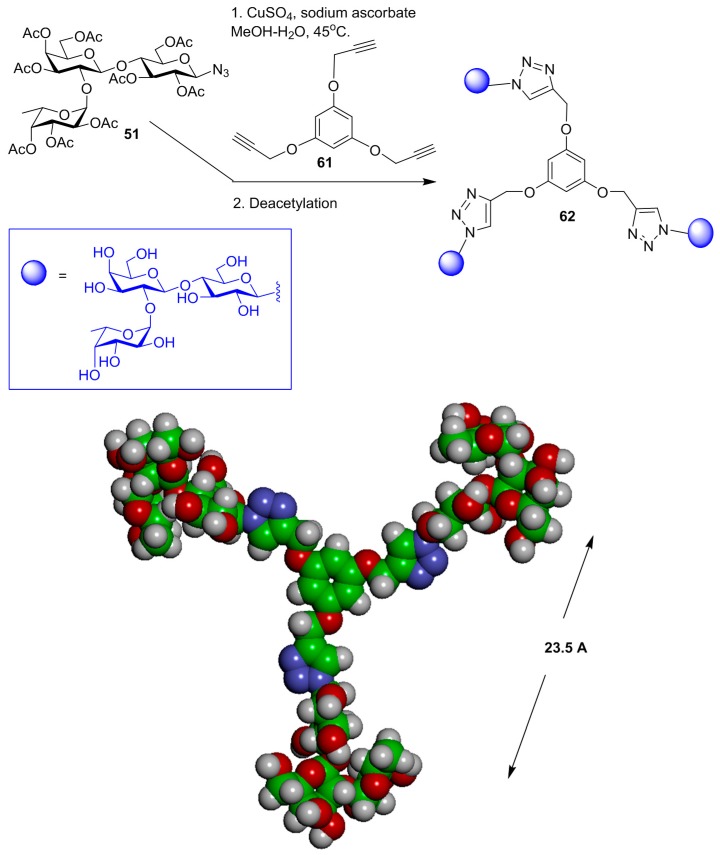
Trivalent 2′-fucosyl lactose-presenting cluster **62** prepared using click-chemistry reaction from **51** and phloroglucinol derivative. In this case, inter-galactoside distances are shorter than in **56**.

The click-chemistry reaction can also be used to give trivalent glycoclusters [89]. Hence, the reaction of 2′-fucosyl azide **51** (Scheme 14) with phloroglucinol derivative **61**, and subsequent protecting group removal gave tritriazole **62**. The distances between galactose residues in **62** was 23.5 Å. This latter compound showed >100-fold selectivity for the chicken galectin-3 relative to the homodimeric galectin-1A, and was 10-fold more potent for the chimera-type protein than the corresponding trilactoside, which was in turn more potent than a related dilactoside. As scaffold with similar degree of rigidity, other choices have been realized, e.g., metal-ion coordinated complexes [112,113] or cyclopeptides [114,115], the latter especially suitable to block binding of galectin-4.

**Scheme 15:**
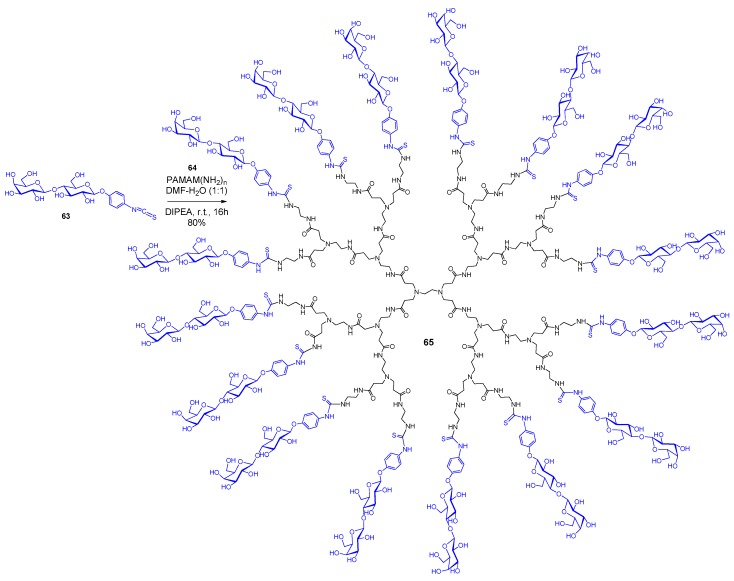
Poly(amidoamine) dendrimer of generation G2 (**65**) incorporating 16 lactoside residues linked through thiourea linkages.

Turning to applications, relative affinity values of glycoclusters for galectins can discriminate between family members, as is the case between galectins-1 and -3. When combining the oligovalent display with a bioinspired substitution at the 3′-position, affinity to galectins-3 and -4 was further enhanced, increasing selectivity against galectin-1 [116,117]. As a different type of application, a synthetic glycocluster has proven as potent tool to determine the stoichiometry of ligand-dependent galectin-3 oligomerization, which depends on a critical length of the *N*-terminal tail on the side of the protein [118,119]. The feasibility to increase the level of branching made it tempting to further raise the valency, entering the realm of glycodendrimers. Poly(amidoamine) starburst (PAMAM) scaffold (Scheme 15) serves as an impressive example. Such a lactosylated glycodendrimer (**65)** up to generation five (G5) (128 lactosides), anchored through an aromatic *p*-isothiocyanatophenyl β-d-lactoside **63** as ligand (Scheme 15), too, reacted differentially with a galactoside-specific plant toxin and galectins-1 and -3 [120]. Of note, the numbers of generation in design progress had a strong impact on reactivity.

In spite of the numerous strategies leading to the syntheses of glycodendrimers, it became obvious that the chemical procedures leading to them would greatly benefit by using core molecules already possessing the highest possible number of exposed surface functionalities. In addition, in order to better control the physicochemical properties of dendrimers, while accelerating the increase of surface groups, it was suggested that different building blocks be used in between each generation. This approach led to the general concept of “onion peel” strategy [121,122,123,124,125] For instance, a glycodendrimer having 18-exposed lactoside residue was readily build-up in a few steps by using a hexaphenylbenzene core such as **69** and a tris(hydroxymethyl)aminomethane (TRIS) precursor suitably substituted (Scheme 7). The synthetic sequence leading to this type of glycodendrimer was initiated by a double Stille coupling reaction involving bis(tributylstannyl)acetylene (**66**) and *para*-iodoanisole (**67**) in the presence of tetrakis(triphenylphosphine)palladium(0) to give the corresponding symmetrical diarylalkyne (**68**) in 71% yield. A [2+2+2] cyclotrimerization of **68** catalyzed by dicobalt octacarbonyl [Co_2_(CO)_8_] and subsequent methyl ether deprotection with BBr_3_ provided rapid access to perhydoxylated **69** in 87% yield. Propargylation of **69** with propargyl bromide and K_2_CO_3_ in DMF 65 °C afforded hexapropargylated ether **70** in 82% yield. Using the orthogonal AB_3_ synthon 2-bromoacetamido-tris[(propargyloxy)methyl]-aminomethane (**71**) gave the targeted 18-mer precursor **72** (79%). The introduction of the modified lactopyranoside termini was efficiently achieved using Cu(I)-catalyzed azide-alkyne [1,3]-dipolar cycloaddition reaction (Scheme 8). To this end, lactosyl azide **4** was “clicked” on either **70** or **72** to afford hexa-lactosylated dendrons (not shown) and 18-mer dendrimer **73**, respectively, in good overall yields after usual transesterification (MeONa in MeOH).

**Scheme 16:**
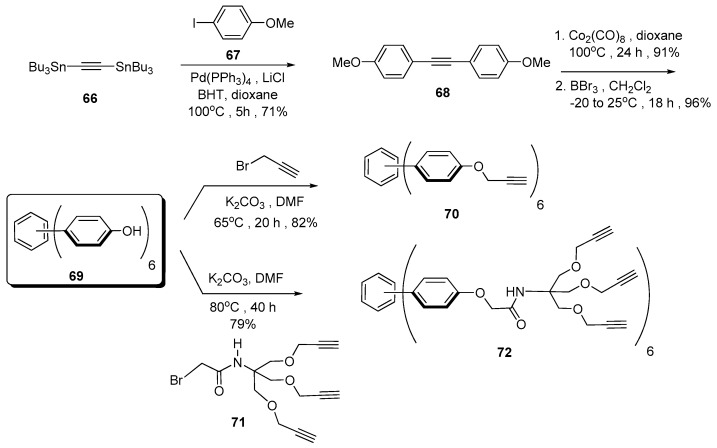
Synthetic procedures leading to hexaphenylbenzene core (**69**) which was either directly propargylated to give **70** or treated with a propargylated TRIS intermediate (**71**) to afford a scaffold harboring 18-propargyl residues (**72**).

Another type of glycodendrimer with ligand radiating from propargylated hexaphenylbenzene cores and containing up to 54 peripheral sugar ligands has been synthesized by the powerful Cu(I)-catalyzed [1,3]-dipolar cycloadditions using both convergent and divergent approaches. An expeditive and systematic route toward the synthesis of innovative glycoclusters and G(1)-glycodendrimers built from circular and aromatic polypropargylated scaffolds have thus been developed (Scheme 16). The multivalent glycoconjugates were obtained with simple and generally high yielding reactions including nucleophilic substitutions to generate synthons and copper-catalyzed azide alkyne cycloaddition reaction to covalently attach the minimally modified galectin ligand as lactoside moieties. Interestingly, the hydrophobic character of the inner aromatic core was counterbalanced by the hydrophilic sugar derivatives, affording fully water-soluble derivatives.

**Scheme 17:**
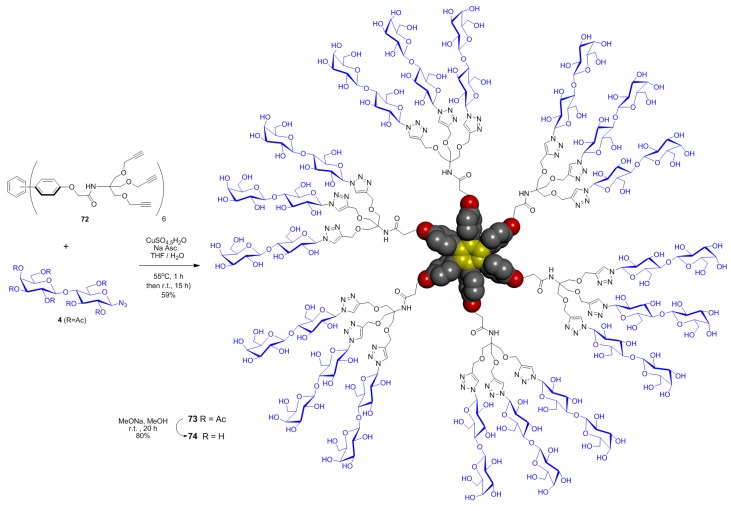
Accelerated synthesis of multivalent lactosides using click chemistry with scaffold **72** and a lactosyl azide (**4**) to give a glycodendrimer with 18-exposed lactoside residues.

**Scheme 18:**
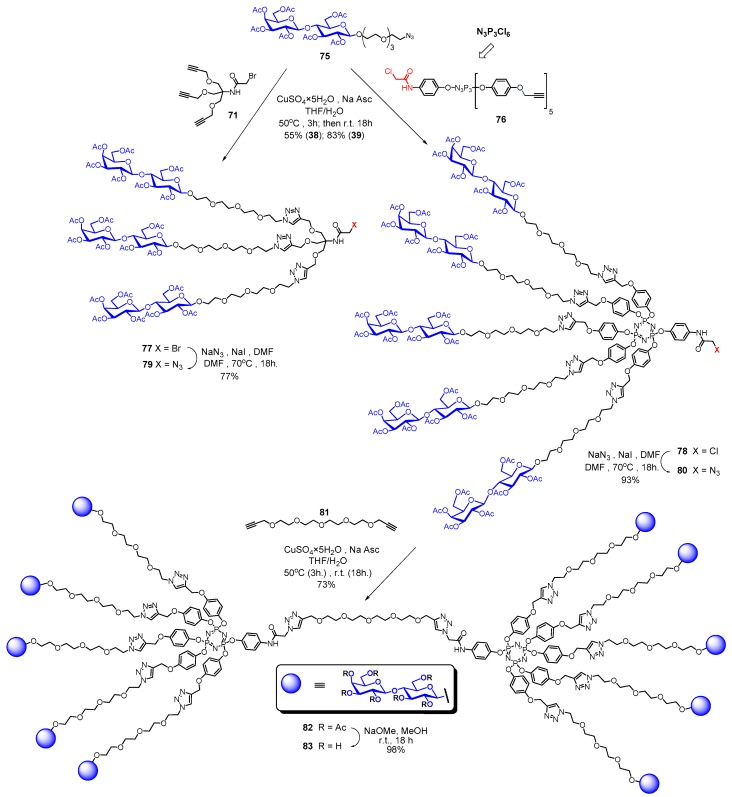
Lactose-presenting glycoclusters with an azide functionality at the focal point were used for the convergent synthesis of highly substituted glycodendrimers **79** (3-mer), **80** (5-mer), and **83** (10-mer) possessing an extended tetraethylene glycol spacer for accessibility to galectins.

Based on hexachlorocyclotriphosphazene (N_3_P_3_Cl_6_) as a central molecule (Scheme 17), which is known to be nearly planar having three up and three down substituents lactosylated dendrons and a dumbbell-shape architecture were obtained (Scheme 18) [126]. In detail, a functionalized AB_3_ trimer, derived from TRIS (**71**, Scheme 16), together with an AB_5_ building block (**76**), derived from the commercially available N_3_P_3_Cl_6_, were used. Lactosylation was achieved using click chemistry with lactosyl azide **75** possessing a tetraethylene glycol spacer. As also used in glycodendrimer synthesis for glycodendrimersome surface programming [127], to give trimer **77** and pentamer **78** in good to excellent yields. Transformation of the bromides at both dendron’s focal point was readily performed by an S_N_2 substitution using sodium azide to afford compounds **79** and **80**, respectively. Pentavalent hypermonomer **80** was then used toward the decavalent dumbbell-shape lactosylated compound **82** by the above double click reaction with dipropargylated tetraethylene glycol **81**. Standard Zemplén de-*O*-acetylation (MeONa, MeOH) generated **79** (3-mer), **80** (5-mer) and **83** (10-mer) in almost quantitative yields in each case.

**Scheme 19:**
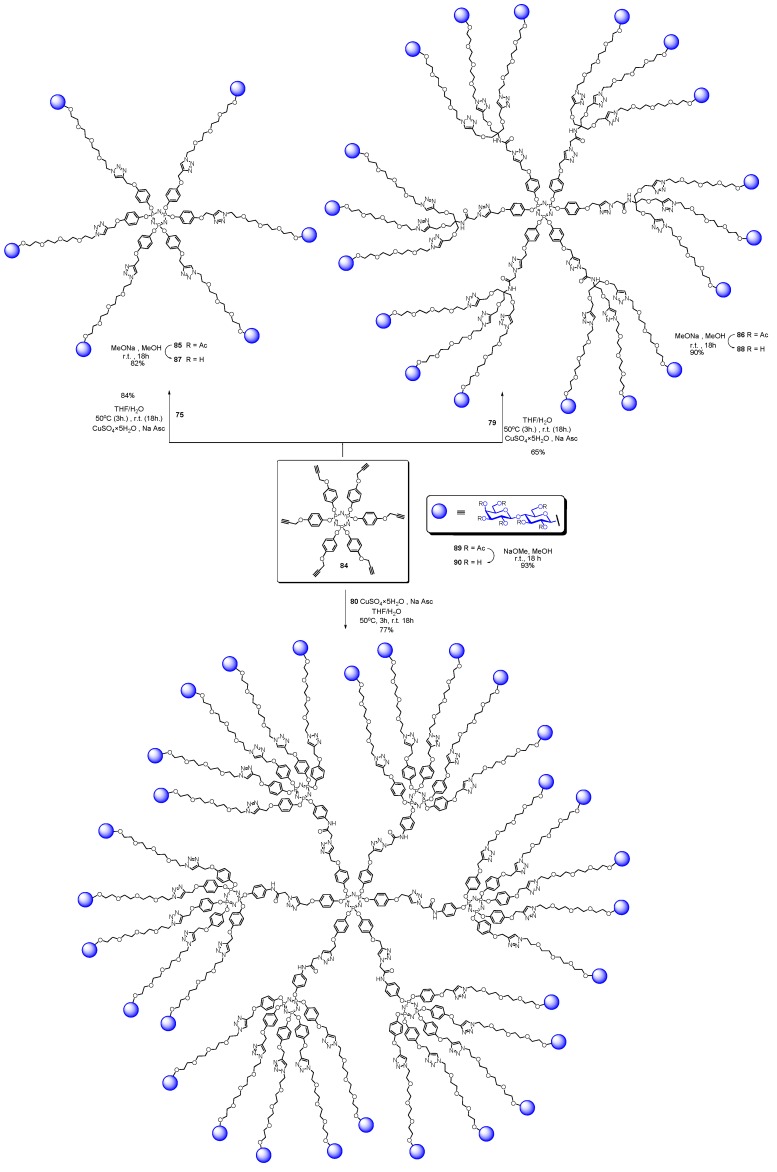
Convergent synthesis of a globular glycodendrimer with six (6-mer, **87**), eighteen (18-mer, **88**), and thirty six (30-mer, **90**) sugar headgroups.

**Scheme 20:**
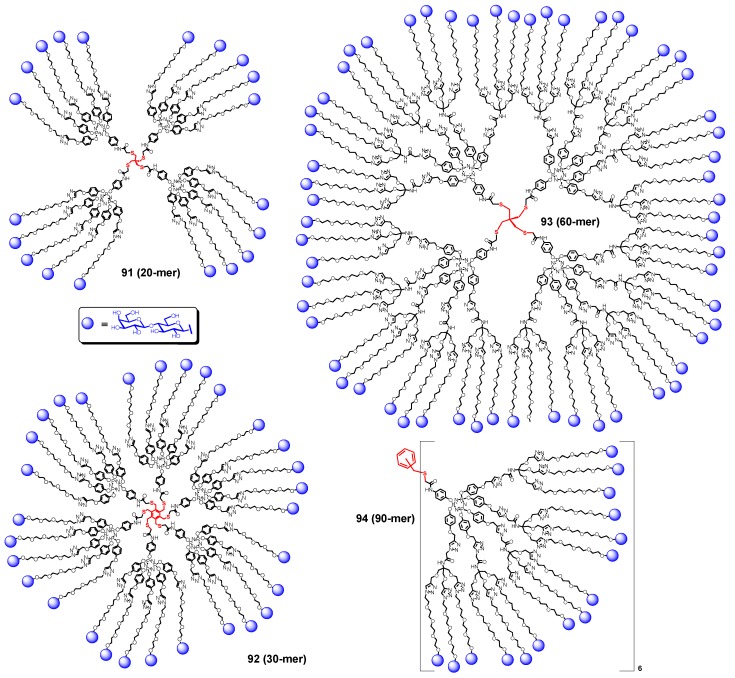
Illustration of the structures of variations of the theme to build glycodendrimers with the same core as in Scheme 18 harboring 20 (**91**), 30 (**92**), 60 (**93**), and 90 (**94**) lactoside residues.

Alternatively, globular-shaped glycodendrimers having six (6-mer, **87**), eighteen (18-mer, **88**), and thirty six (36-mer, **90**) were constructed using the extended lactoside residues (Scheme 19) [126]. Thus, using a hexapropargylated cyclotriphosphazene derivative (**84**) and the copper-catalyzed [1,3]-dipolar cycloaddition together with azide **75**, the three compounds were prepared in 82%, 65%, and 77% yields, respectively. After Zemplén trans esterification, the unprotected lactosylated dendrimers were obtained. Interestingly, high-field ^1^H-NMR spectra clearly probed completion of the multiple click processes, as judged by the complete disappearance of the critical alkyne protons at δ 2.64 ppm. In addition, all the relative integrations of each triazole protons around δ 8 ppm were in perfect agreement with those of the newly formed internal region (18H *vs.* 6H, respectively for 18-mer **88**). Moreover, MALDI-TOF experiments afforded typical isotopic patterns for **90** with the expected molecular weight signal ([M + Na]^+^ adduct) at *m*/*z* 23,750 Da. Using an analogous strategy, lactodendrimers harboring 20-mer, 30-mer, 60-mer, and finally 90-mer were also constructed (Scheme 20) starting from thiolated pentaerythritol and hexasubstituted benzene derivatives [126]. The above structures were shown to have low polydispersity PDI (Mw/Mn) (<1.08) with molecular weight ranging from 5 kDa to more than 75 kDa [126].

The size of the glycodendrimers containing both the acetylated and the deprotected functionalities, and more particularly their solvodynamic radii, was estimated by pulsed-field-gradient stimulated echo (PFG-STE) NMR experiments using bipolar pulse pairs longitudinal-eddy-current delay (BPP-LED) in CDCl_3_ and D_2_O, respectively, at 25 °C, as described in detail [126]. Diffusion values were determined by the average of individual values corresponding to the decay of the signal intensity of different protons located at different level in the molecule. The resulting measurements were consistent with spherical and unimolecular character of the glycodendrimers. The Stokes–Einstein equation directly yielded the corresponding solvodynamic diameters using viscosities determined for pure deuterated solvents. As expected, diffusion data revealed the increase of solvodynamic diameters as the number of peripheral epitopes was enhanced, under similar solvent conditions, showing dendrimers with solvodynamic diameters ranging from 2.6 to 10.1 nm. Systematic monitoring of bioactivity with the CRD of galectin-3, physiologically a product of proteolytic processing that removes the N-terminal tail (please see Figure 3), revealed ligand property for all compounds. As noted above for starburst glycodendrimers, the relative potency per sugar unit was highest at low to medium level of valency, here for the dumbbell-shapes decavalent **83** (r.p./sugar = 53) of a diameter of 5.3 nm [126]. These examples document the ingenuity of chemical design to reach the level of giant clusters with a core. To take design even further into the direction of engineering vesicle-like structures, a different strategy has been successfully implemented.

**Scheme 21:**
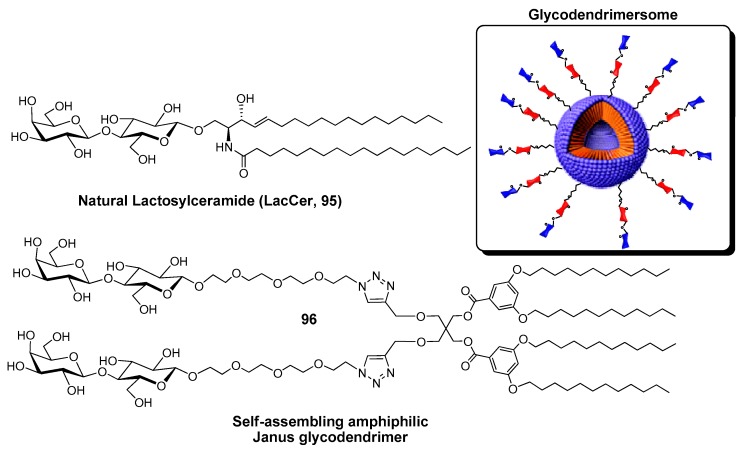
Typical structure of a natural glycolipid (LacCer) compared to a member of the recently synthesized family of self-assembling amphiphilic Janus glycodendrimers capable of forming stable liposomes (glycodendrimersomes).

The way (glyco)lipids self-organize into monolayers or liposomes has been equally efficiently achieved with amphiphilic Janus glycodendrimers (for a comparison of a glycolipid and the synthetic analogue, please see Scheme 21) [127]. The products are glycodendrimersomes or aggregates of other types of design such as cubosomes with fully programmable properties in size and surface properties [127,128]. For example, ligand density on micro- and macroscales can be changed in a defined manner, as branching in glycodendrimers is. These products are ideal for interaction studies with lectins. Here, the influence of any parameter on both sides on aggregation can be examined. These assays with human galectins have already answered fundamental questions on galectin functionality in *trans*-interactions and as a function of carbohydrate density and headgroup display [129,130,131]. Galectin-1’s design as non-covalently associated homodimer (please see Figure 3) appears more suited for cross-linking counter receptors on the surface than to act as bridge between glycodendrimersomes, as indicated by comparative analysis with engineered variants that have covalent connections between the two CRDs [129]. Fittingly, atomic force measurements have revealed a comparatively low rupture of 37 ± 3 pN at a loading rate of 3 nN/s, when galectin-1 interacted with *N*-glycans of the glycoprotein asialofetuin [132]. An emerging aspect for endogenous lectins is the occurrence of genetic polymorphisms. It is a pertinent question whether they have pathophysiological significance. As biomedical effector, it is noteworthy that this lectin can have anti- and pro-inflammatory/tumoral activities depending on the context, for example stimulating tissue invasion (glioblastoma) or cartilage degeneration (osteoarthritis) or acting as negative regulator in chronic inflammation or carcinoma cell cycle progression [133,134,135,136,137,138,139].

For the first time, a functional correlation of a single nucleotide polymorphism in a CRD of a galectin could be discerned with this assay system, here for galectin-8’s F19Y variant whose expression is strongly associated with occurrence of rheumatoid arthritis and early age at disease onset [130,131,140]. This approach thus takes activity testing to the level of vesicle-like structures, and the recently accomplished hybrid formation with bacterial membranes [141] let the perspective become envisioned to combine natural glycoconjugates with these synthetic glycodendrimers to tailor surface properties in a so far unsurpassed manner according to the biomedical question to be addressed. Design of monolayers with the same toolbox will flank the analysis of aggregation (*trans*-interaction). In such a setting, *cis*-bridging can be monitored, as exemplified by monitoring galectin-1/ganglioside GM1 interplay [142].

## 6. Conclusions

The increasingly unraveled physiological significance of carbohydrate-lectin recognition poses challenging questions on the nanometric characteristics underlying the specificity and selectivity of the functional pairing. As the example of the adhesion/growth-regulatory galectins attests, delineating structure-activity relationships with respect to the intrafamily divergence in CRD sequence and its spatial presentation requires sophisticated tools and transsectional cooperation. Here, the individual parts of the toolbox provided by synthetic chemistry are invaluable sensors, e.g., to determine the capacity for multivalent binding and induction of cell signaling that may cause harmful effects. Actually, glycoclusters had been helpful to ascertain a reduction in cross-linking, leading to undesired T cell activation, within the process of engineering an antiviral lectin for further testing of its potential for therapeutic application [143]. Along the route to obtain insights into the biofunctionality of the nanometric features, bringing test systems for activity assessment of the synthetic products as close as possible to the *in vivo* conditions, e.g., by testing their effect on lectin binding to tissue sections [144], will team up with the design of vesicle-like structures with increasing degree of resemblance to cell surfaces. By rational tailoring on both sides, *i.e.*, the ligand and the lectin, as hypothesis-driven process, these advances bring understanding how endogenous lectins ‘read’ sugar-encoded information into reach.

The monovalent [145,146] as well as the multivalent glycomimetic lactoside derivatives presented herein offer several avenues to identify high affinity galectin-selective antagonists for the treatment of various diseases. Nanometric glycodendrimers in particular, with their size ranging from 2 to 10 nm and their high water-solubility, constitute a valuable alternative to other types of insoluble nanoparticles [147]. Of great interest in this regard, is the new family of glycodendrimersomes described in the last section which are also nanometric (100–150 nm, for liposomes) or 100–200 μm when forming giant vesicles. These biomimetics of cell membranes have the fortuitous possibility to adapt their flexible inter-carbohydrate distances in response to the galectin receptors they encounter, thus adding an additional advantage over other nanoparticles.

## Figures and Tables

**Table 1:** Amino acids of the CRDs of six galectins directly interacting with lactose residues are shown in red, and amino acids that interact with lactose via water-mediated hydrogen bonds are shown in blue. Comparison with galectin-1 (PDB-1GZW), galectin-2 (PDB-1HLC), galectin-3 (PDB-1KJL), galectin-4 (PDB-1X50), galectin-7 (PDB-4GAL), and galectin-8 (PDB 3AP9). Amino acids letter codes are indicated in parentheses for Gal-3 [26].

CRD-Subsite	Galectin-1	Galectin-2	Galectin-3	Galectin-4C	Galectin-7	Galectin-8N
A	Ser29	Gly30	Arg144 (R)	Ser220	Arg31	Arg45
B	Val31	Val32	Ala146 (A)	Ala222	His33	Gln47
C	Asn33	Asn34	Asp148 (D)	Asn224	Asn35	Asp49
D	Gly35	Gly36	Gln150 (Q)	Lys226	Leu37	Gln51
E	-	-	-	-	-	Arg59
F	His44	His45	His158 (H)	His236	His49	His65
G	Asn46	Asn47	Asn160 (N)	Asn238	Asn51	Asn67
H	Arg48	Arg49	Arg162 (R)	Arg240	Arg53	Arg69
I	His52	-	-	-	-	-
J	Asp54	Gln52	Glu165 (E)	-	-	-
K	Asn61	Asn58	Asn174 (N)	Asn249	Asn62	Asn79
L	Trp68	Trp65	Trp181 (W)	Trp256	Trp69	Trp86
M	Glu71	Glu68	Glu184 (E)	Glu259	Glu72	Glu89
N	Arg73	Arg70	Arg186 (R)	Lys261	Arg74	Ile91
O	-	Arg120	Ser237 (S)	Gln313	Gly124	Tyr141
